# Systematic Review of Efficacy and Safety of Newer Antidiabetic Drugs Approved from 2013 to 2017 in Controlling HbA1c in Diabetes Patients

**DOI:** 10.3390/pharmacy6030057

**Published:** 2018-06-27

**Authors:** Sivanandy Palanisamy, Emily Lau Hie Yien, Ling Wen Shi, Low Yi Si, See Hui Qi, Laura Soon Cheau Ling, Teng Wai Lun, Yap Nee Chen

**Affiliations:** 1 Department of Pharmacy Practice, International Medical University, Kuala Lumpur 57000, Malaysia; 2 School of Pharmacy, International Medical University, Kuala Lumpur 57000, Malaysia; emilie_0705@hotmail.com (E.L.H.Y.); lingwenshi@hotmail.com (L.W.S.); yisilow95@gmail.com (L.Y.S.); huiqi0325@gmail.com (S.H.Q.); laura_son@hotmail.com (L.S.C.L.); ashteng.96@gmail.com (T.W.L.); alicemuimail@gmail.com (Y.N.C.)

**Keywords:** diabetes mellitus, antidiabetic agent, efficacy, safety, glycosylated hemoglobin

## Abstract

Type 2 Diabetes Mellitus (T2DM) is the most common form of diabetes mellitus and accounts for about 95% of all diabetes cases. Many newer oral as well as parenteral antidiabetic drugs have been introduced in to the market in recent years to control hyperglycemic conditions in diabetes patients and many of these drugs produce potential side effects in diabetes patients. Hence, this systematic review was aimed to analyze and compare the efficacy and safety of oral antidiabetic agents in controlling HbA1c in T2DM patients, that were approved by the United States-Food and Drug Administration (US-FDA) from 2013 to 2017. All randomized controlled, double-blind trials published in English during the search period involving the newer antidiabetic agents were selected. In the outcome assessment comparison, semaglutide demonstrated the highest efficacy in lowering HbA1c, with a 1.6% reduction (*p* < 0.0001) when given at a dose of 1.0 mg. The safety profile of all the agents as compared to placebo or control were similar, with no or slight increase in the occurrence of adverse events (AEs) but no fatal reaction was reported. The most common AEs of all the antidiabetic agents were gastrointestinal in nature, with several cases of hypoglycemic events. However, among all these agents, semaglutide seems to be the most efficacious drug to improve glycemic control in terms of HbA1c. Alogliptin has the least overall frequency of AEs compared to other treatment groups.

## 1. Introduction

Type 2 Diabetes Mellitus (T2DM) is the most common form of diabetes mellitus where it accounts for 90–95% of all diabetes cases [1,2,3]. It was described as a metabolic syndrome in 1988 and is characterized by hyperglycemia and defects in secretion and sensitivity of insulin [1,4]. T2DM occurs in a progressive nature by starting with insulin resistance, declining insulin secretion followed by the failure of pancreatic beta-cell [1,3,4]. The microvascular and macrovascular complications from T2DM also detrimentally affect the patients’ quality of life and often lead to death. The most common complications reported in T2DM are cardiovascular disease, neuropathy, nephropathy and retinopathy [1,5,6]. According to the American Diabetes Association (ADA), the management of T2DM requires multiple risk-reduction strategies and demands ongoing medical care which lead to medical and economic burdens. There are approximately about 190–325 million people that will be estimated as suffering from diabetes in the next 25 years globally [1,3,4]. The risk factors of getting T2DM are mainly due to genetic susceptibility and environmental influences [1,7,8]. The therapeutic goals of T2DM are to achieve and maintain glycemic targets, mitigate hypoglycemia and reduce the development of complications that lead to morbidity and mortality, especially cardiovascular disease [1,4]. Despite the increasing prevalence worldwide over the past three decades, to date, no cure has been found and widely used for the disease. Metformin, a biguanide which reduces insulin resistance remains as the recommended first line medication for T2DM [9,10,11]. Despite the advancements in treatment for T2DM, the majority of patients do not achieve their target control of glycosylated haemoglobin (HbA1c) with a reporting failure rate approximately 63% [1,3,4]. Thus, dietary advice will be individualized as an adjunct to the treatment of T2DM to improve glycemic control. Nevertheless, the continuous efforts in developing novel treatment modalities led to the introduction of new medications in the past five years such as glucagon-like peptide 1 agonist, dipeptidyl peptidase-4 (DPP-4) inhibitors, and sodium-glucose cotransporter-2 inhibitors. These medications are selected based on the patient variables and their clinical data.

## 2. Method

A systematic review is where the latest and most complete information can be found, and it acts as a crucial tool for healthcare professionals to evaluate the reliability and clinical significance of a topic or intervention. A systematic review can convey the summarized information and comprises a comprehensive and systematic searching of the literature, and reducing the selection bias that could be found in a review. It includes a synthesis of results from previous research, where it can be accompanied by meta-analysis which involves analyzing the data and combining them into an average result. The synthesis is then used to make conclusions and give recommendations.

### 2.1. Outcome Assessment

The primary outcomes of this review were the efficacy and safety of oral antidiabetic drugs that are commonly used for the treatment of hyperglycemic conditions in T2DM patients. To assess the efficacy of oral antidiabetic drugs measured by the reduction in blood glucose level, HbA1c was considered for this review. The drug safety includes the reduction in adverse drug effects/adverse events compared with previously introduced drugs in the market that had been approved by US-FDA.

### 2.2. Data Extraction

The data extracted for this review is mainly on the oral antidiabetic drugs used in the treatment of Type 2 Diabetes Mellitus. Preferred reporting items for systematic reviews and meta-analysis (PRISMA) was used for selection of articles and reporting of reviews that evaluating randomized controlled trials. The main sources of data used were PubMed, Nvivo, Mendeley, Evernote, CiteUlike, Biohunter, Delvehealth, Scicurve, and Google Scholar, etc. Articles on Type 1 diabetes mellitus and animal studies were excluded. The anti-diabetic drugs included in our studies are those that were approved by US-FDA from the year 2013 to 2017, as according to Centre Watch. There are altogether eight drugs approved in the search period, that are reviewed and 72 articles of randomized controlled trails (RCTs) were collected. All the authors independently extracted the relevant information from the RCT studies that fulfilled our inclusion criteria and any disagreements were resolved with consensus. The information extracted included the trial phase, region, conditions of subjects (mean age, mean blood glucose concentrations, mean HbA1c, comorbidities) and the outcome measures. This information was gathered and summarized into paragraphs, introducing each antidiabetic drug comprehensively.

## 3. Results and Discussion

### 3.1. Mechanism

Among the eight drugs that were approved by US-FDA during the search period, Canagliflozin, Dapagliflozin and Empagliflozin act as sodium-glucose transporter-2 (SGLT-2) inhibitors; Albiglutide, Dulaglutide, Lixisenatide and Semaglutide act as glucagon-like peptide-1 (GLP-1) receptor agonists, and the Alogliptin act as dipeptidyl peptidase-4 (DPP-4) inhibitor. All these drugs work in three different mechanism of action to reduce the hyperglycemic condition in T2DM patients. The mechanisms of action of all eight drugs are discussed in detail.

#### 3.1.1. Canagliflozin

Canagliflozin is an SGLT-2 inhibitor used to treat Type 2 Diabetes Mellitus in many countries such as those in North-America, Europe, Latin America and Asia-Pacific [12]. It acts differently compared to other antidiabetic agents by exerting its effect primarily on renal glucose handling. Canagliflozin lowers blood glucose and improves glycemic control through an insulin-independent mechanism. It works by stimulating the urinary excretion of glucose through inhibiting renal glucose absorption [13]. This advantage helps SGLT-2 inhibitors to be used in dual and triple therapy regimens because it has a lower risk of getting hypoglycaemia [14,15]. The renal glucose reabsorption is facilitated by SGLT-2, which is mostly expressed in S1 segment of the proximal tubule. It works by reabsorbing filtered glucose until the filtered load goes beyond the capacity of the transporters [16]. This reaction leads to osmotic diuretic effects and caloric loss which eventually causes reduction in body weight, and thus helps in obese diabetic patients [14,16].

#### 3.1.2. Alogliptin

Alogliptin benzoate is highly selective [17]. It inhibits DPP-4 which is the main enzyme which rapidly degrades the incretin hormones such as glucose-dependent insulinotropic peptide (GIP) and GLP-1. GIP is produced at normal levels with disabled glucose-lowering action in patients with T2DM. Despite decreased secretion, GLP-1 still keeps its glucoregulatory activity. Therefore, concentrations of active incretions such as GIP and GLP-1 increase due to the inhibition of DPP-4 activity [18]. These two incretins can activate the pancreatic β cells to secrete insulin, and also promote proliferation of the beta-cell and cytoprotects to improve homeostasis of glucose. Alogliptin is prescribed to be combined with metformin even though they have different mechanisms of action in improving glucose metabolism [17]. Moreover, alogliptin can reduce inflammation and thereby perform anti-atherosclerotic effects by inhibiting the monocyte activation/chemotaxis [19]; it prevents migration of monocytes and actin polymerization via Rac-dependent mechanism [20], and can act by inhibiting the inflammation in diabetic apoE-deficient in animals [21].

#### 3.1.3. Dapagliflozin

Dapagliflozin is an orally active selective SGLT-2 inhibitor. It improves blood glucose control in patients with T2DM by inhibiting SGLT-2 in the renal proximal tubule where most of the reabsorption of filtered glucose occurs, thus eliminating blood glucose through urine [22,23,24]. It improves insulin sensitivity in the body and lowers fasting plasma glucose (FPG) through inducing glycosuria [24,25,26]. Dapagliflozin is used as an adjunct to diet and exercise to improve glycemic control in T2DM patients. The initial dose is 5 mg once daily and can be increased to 10 mg once daily in patients requiring additional glycemic control [25]. A study by the FDA showed that dapagliflozin significantly reduced body weight and improved HbA1c and FPG compared to placebo [27].

#### 3.1.4. Empagliflozin

Empagliflozin is a potent and selective inhibitor of SGLT-2. It is proven to improve glycemic control and reduce blood pressure and body weight [28,29]. It works by increasing the urinary glucose excretion (UGE) in diabetes patients by inhibiting SGLT-2-mediated renal glucose reabsorption in the proximal tubule of nephrons [30,31,32,33]. The mechanism of action of empagliflozin is independent of beta-cell function and insulin secretion [32]. It is suggested to be used as monotherapy or as adjuvant [30,31] to the patients, and is safe to be combined with exogenous insulin [28]. Empagliflozin can be used in improving glycemic control with a low risk of hypoglycaemia [28]. Significant reductions in glycated hemoglobin (HbA1c), fasting glucose, blood pressure, weight and the frequency of symptomatic hypoglycemic events can be seen in empagliflozin [30]. It also reduces insulin dose requirements and alleviates weight gain induced by insulin besides improving glucose control [28]. It also reduces glycemic variability and oxidative stress.

#### 3.1.5. Albiglutide

Albiglutide is a long-acting GLP-1 receptor agonist [34]. It helps to improve glycemic control in patients with T2DM [1,34,35]. Albiglutide is a subcutaneous injection that comes as a lyophilized powder in a prefilled dosing pen and it must be reconstituted before use and administered by weekly injection [1,34]. It can be indicated as monotherapy or combination therapy to control blood glucose for the management of T2DM [1,34,35]. However, albiglutide is not indicated for diabetic any patient who is inadequately controlled with diet and exercise [1,34,35]. The biological half-life of albiglutide is 5 days. This long half-life results in resistance on degradation of DPP-4 fused to albumin which allows albiglutide to be administered by weekly injection [34]. According to the HARMONY phase 3 programme, albiglutide is safe and well-tolerated in patients with T2DM [1,34]. A missed dose of albiglutide should be administered within 3 days [34].

#### 3.1.6. Dulaglutide

Dulaglutide is a long acting GLP-1 receptor agonist which is administered through subcutaneous injection [36,37,38,39,40,41,42]. The approved dosage of Dulaglutide in type 2 diabetes mellitus are 0.75 mg as well as 1.5 mg once weekly in USA and European Union and 0.75 mg in Japan [36,37,41,43]. Molecule of dulaglutide consists of two identical GLP-1 analogues, joined by disulphide bond, each containing an N-terminal GLP-1 analogue sequence covalently linked by a small peptide to a modified human immunoglobulin G4 heavy chain [39,40,42,44,45]. Structural modification on Dulaglutide causes it to stabilize against degradation by DPP-4, results in the rise of solubility of the peptide and its duration of activity as well as reduction in the immunogenic potential [39,41,42,43]. Half-life of dulaglutide is approximately 5 days thus resulting it to be an appropriate drug for once-weekly dosing [39,43]. Dulaglutide mimics the effects of endogenous GLP-1 which is released from intestinal L-cells in the presence of food. It stimulates the release of glucose-dependent insulin, suppressing the secretion of glucagon, delaying gastric emptying as well as reducing food intake to facilitates glucose metabolism [41,42,43,46].

#### 3.1.7. Lixisenatide

Lixisenatide is a selective GLP-1 receptor agonist used for treating Type-2 DM [47]. In a dose ranging study in T2DM patients who are insufficiently controlled with metformin, a 20 μg once-daily lixisenatide demonstrated with greatest efficacy-to-tolerability ratio [47]. Lixisenatide produces its effects by direct stimulation of GLP-1 receptors located in the pancreatic islet β-cells, restoring the glucose-sensitive stimulation of insulin secretion, thereby promoting glucose disposal to normalization of glucose level [48]. Besides, lixisenatide is beneficial in improving control of post-prandial glucose (PPG) excursions by delaying gastric emptying and increasing the meal-derived glucose absorption time [49,50]. In addition, lixisenatide treatment has led to the reduction of FPG in a dose-dependent manner by stimulating the release of glucose-sensitive insulin [51].

#### 3.1.8. Semaglutide

Semaglutide is a GLP-1 receptor agonists administered by subcutaneous injection [52]. It acts as an adjunctive therapy in the treatment of patient with T2DM with a controlled diet and exercise to improve the glycemia control [52,53,54,55]. The approved therapeutic dose of semaglutide are 0.5 mg and 1 mg. It must be administered as once weekly on the same day every week and either same or different time of the day [55]. Semaglutide is not recommended for patient with type 1 diabetes or diabetic ketoacidosis [52,53].

It is an analogue of native human GLP-1. The high affinity binding of albumin gives long plasma half-life which allow it to be administered as weekly injection. Its biological half-life is 168 h [53]. Semaglutide improve the glycemic control through several mechanisms by increasing the secretion of insulin, slow gastric emptying and lower the fasting and postprandial glucose. GLP-1 increase the insulin secretion when glucose is ingested [53]. At the same time, it slows gastric emptying to inhibit the release of post-glucagon and increase satiety. The unique of semaglutide is because of its structural modifications where generate a durable stability against DPP-4 [53]. This modified chemical structure of semaglutide provides extended half-life and improve the quality of life as well as compliance of patient [52,53].

### 3.2. Efficacy

The individual drug’s efficacy in controlling HbA1c level in diabetes patients and their safety profile were compared with every individual drugs those that are approved during the search period from 2013 to 2017.

#### 3.2.1. Canagliflozin

The efficacy of canagliflozin has been evaluated since the year it was introduced. The minimum effective dose of canagliflozin was 30 mg, for lowering of random blood glucose, fasting blood glucose and 24-h plasma glucose levels [16]. The reduction in blood glucose level was dose-dependent and the clinically significant dose of canagliflozin was found to be 100–300 mg [13,14,15,16]. Placebo-subtracted WMDs (%) of HbA1c were −0.63 and −0.80 for canagliflozin 100 and 300 mg, respectively from baseline to week 26. [56,57,58,59,60,61,62]. A starting dose of 100 mg was recommended as it was found to be safe for patients of all ages, especially in patients aged 75 and above [59]. In term of HbA1c lowering effect, canagliflozin was found to be non-inferior to metformin at dose of 100 mg and 300 mg, but a combination of metformin and canagliflozin could provide statistically significant reductions in HbA1c compared to metformin or canagliflozin alone [62]. However, similar to other fixed-dose combinations, this combination also showed sub-additive efficacy. In another randomized, double blind phase 3 study, canagliflozin demonstrated to produce equal effect as that of sitagliptin at a dose of 100 mg and more effects at 300 mg [57].

Like other SGLT-2 inhibitor, canagliflozin also associated with the reduction in body weight [12,13,14,15,16,21,56,58,59,60,61,63,64,65]. As with the reduction in blood glucose level, the weight loss was also dose-dependent. Canagliflozin dose of over 100 mg were linked to 1–1.5 kg weight reduction, with a stable body weight reduction of 2–4% in long term studies [16]. In general, canagliflozin was shown to be effective alone and could be combined with other antidiabetic agents such as metformin, sitagliptin, pioglitazone, sulphonylurea and insulin [12,14,56,57,62,63]. It was also found to be equally effective in patients with different stages of T2DM of different races, indicating its therapeutic potential for every diabetic patient [14,58].

#### 3.2.2. Alogliptin

Alogliptin, a DDP-4 inhibitor has advantage over other antidiabetic agents as it does not produce weight gain [66], and its ability to inhibit atherosclerosis and inflammation [19]. Alogliptin helps to improve glycemic control in adults with T2DM together with diet and exercise plan [18]. It can produce rapid and sustained DPP-4 inhibition and improve both postprandial and fasting plasma glucose. After 12 to 26 weeks of treatment in adult patients, the HbA1c has been reduced by approximately 0.5% to 1% [18]. However, many studies proved that it brings better glycemic control when combined with other non-DPP-4 inhibitor antidiabetic agents, instead of alogliptin alone [18,67]. A primary analysis had demonstrated the superiority of combination of alogliptin and metformin OD over the use of alogliptin alone. The analysis also revealed the non-inferiority of single dose and double dose daily use of alogliptin/metformin [17,67]. At every time point, the mean HbA1c was found to be lower in these two dosing, but the lowering effect is not seen with alogliptin alone. These dosing were reported to have similar reduction effect on HbA1c at 20 weeks of assessment [17]. At the end of treatment, the proportion of patients with reduction in HbA1c to below 7% for patients on alogliptin alone, on alogliptin/metformin OD and alogliptin/metformin BD dosing were 4.8%, 35.0% and 34.3% respectively. It was also found out that combination of alogliptin and metformin achieved a better reduction in the mean change of FPG, with results of −7.6 mg/dL and −18.2 mg/dL respectively when compared with aloglitin alone which caused a 7.4 mg/dL increase [17].

#### 3.2.3. Dapagliflozin

After 25-week of treatment with dapagliflozin, mean amplitude glycemic outcome (MAGE), standard deviation of mean blood glucose (SDMBG), the incremental area under curve (AUC) of blood glucose above 10.0 mmol/L, the 24-h mean blood glucose (MBG) and the AUC above fasting plasma glucose (FPG) have been improved [22]. Dapagliflozin increases cardiovascular disease benefits by reducing hyperglycemia, systolic and diastolic blood pressure, body weight and atrial natriuretic peptide (ANP); it also decreases serum uric acid and rise in parathyroid hormone levels [22,23]. In contrast to other antidiabetic agents that could cause weight gain, dapagliflozin causes weight loss and reduces BMI [22,23]. It also reduces glycemic variability and the reduction of oxidative stress [22]. Another study showed that dapagliflozin is more superior in decreasing HbA1c, FPG and 2-h post-prandial plasma glucose as compared to other antidiabetic agents [68]. Also from this study it clearly revealed that dapagliflozin reduces subcutaneous and visceral abdominal adipose tissue and increases calorie expenditure through urine. It increases osmotic diuresis and natriuresis which in turn reduce plasma volume [25]. However, there is no hypotension or dehydration been reported. Dapagliflozin can also be used to prevent the development of pre-diabetes. It can be as an adjunct therapy in patients on insulin as it decreases the dose of insulin through the means of improving insulin sensitivity. There are numerical changes in high density lipoprotein (HDL) level as clearly stated in the study done by Araki, et al. [25]. Besides, the dapagliflozin also increases the fasting plasma glucagon and reduces fasting plasma insulin, without causing hypoglycaemia [22,26].

Moreover, the dapagliflozin can effectively reduce HbA1c and fasting blood glucose especially in patients with mild to moderate renal impairment. Dapagliflozin is shown to be effective in reducing T mg (renal glucose reabsorption). It decreased the threshold of glucose excretion in urine, thus more glucose can be excreted. The study also revealed that dapagliflozin works at any stage of T2DM owing to its independency on severity of insulin resistance and beta cell failure [69]. Moreover, study from Lambers Heerspink, et al. [70] proved that dapagliflozin increased plasma renin activity and serum aldosterone concentration. It also increases the N-terminal pro b-type natriuretic peptide (NT-pro-BNP) and Brain Natriuretic Peptide (BNP) which causes the reduction of plasma volume and diuresis respectively [70].

#### 3.2.4. Empagliflozin

Empagliflozin is a SGLT-2 inhibitor that is effective in reducing blood glucose level. A reduction in mean blood glucose levels was shown since the first day of administration of empagliflozin, and greater reduction was observed with empagliflozin 25 mg as compared to empagliflozin 10 mg [32]. Empagliflozin 10 mg and 25 mg reduced the time of patient suffering from hyperglycemia (glucose ≥ 180 mg/dL), maintained the patient on normoglycemia (glucose ≥ 70 to <180 mg/dL) with a slight increase in the chance of getting hypoglycemia (glucose < 70 mg/dL) [32,71]. Empagliflozin reduces inflammation and oxidative stress in hepatic, renal and cardiac tissues through some experimental studies thus it is presumably would have a protective effect on the cardiovascular system [71]. This would be highly advantageous for diabetic patients with increased cardiovascular risk, and also Congestive Heart Failure (CHF) patients who need frequent administration of diuretic agents. However, the efficacy of empagliflozin on diabetic patients with CHF should be monitored again as there is possibility of excess intravascular fluid depletion.

#### 3.2.5. Albiglutide

The efficacy of albiglutide on the changes in HbA1c was evaluated by a 52 weeks randomized and placebo-controlled study [60,72]. A once weekly injection of albiglutide 30 mg, albiglutide 30 mg with up titration to 50 mg once weekly or placebo were randomly assigned to eligible patients [60,72]. A reduction of HbA1c was shown in both treatment groups with the administration of albiglutide 30 mg and 50 mg while increase of HbA1c was seen in the placebo group [35,72,73]. Change of FPG from baseline over time, HbA1c targets and time to hyperglycemic rescue were consistent with the result of HbA1c [73]. However, there is no significant reduction in the body weight between placebo group and treatment group [35,72,73]. The study reported that number of rescue therapy required was significantly higher in placebo patients compared to albiglutide 30 mg and 50 mg group with the probability of 55.7%, 21.7% and 18.6% respectively [73]. Albiglutide treatment had shown steep decline of HbA1c from baseline up to 2 weeks and maintained up to 52 weeks due to its sustained effect [51]. A greater reduction in HbA1c was also observed in albiglutide treatment when compared with exenatide and lixisenatide [1,35,72,73].

#### 3.2.6. Dulaglutide

Throughout a 24 weeks’ phase III, randomized, double-blind and placebo-controlled study, there was a 1.4% reduction of HbA1c from baseline in Dulaglutide 1.5 mg group as compared to −0.1% in placebo group. At 24 weeks, 55.3% participants from dulaglutide group and 18.9% participants from placebo group reached the HbA1c target of <7.0% while 40% and 9.4% from respective group achieved HbA1c <6.5% [39]. According to an analysis combining data from three phase III studies in the Japanese T2DM patients receiving dulaglutide, dulaglutide treatment was found to have significant difference in HbA1c reduction from baseline among the four subgroups (young/low BMI, young/high BMI, elderly/low BMI, elderly/high BMI) with smallest reduction in the young/high BMI subgroup and highest reduction in elderly/high BMI subgroup [37].

Across a six head-to-head phase 3 clinical trials study [38], the extent of reduction in HbA1c was found to depend on the dose of dulaglutide used. Dulaglutide 1.5 mg shown greater reduction from baseline in HbA1c than dulaglutide 0.75 mg. HbA1c reduction for dulaglutide 1.5 mg and 0.75 mg in monotherapy was 0.78% and 0.71% respectively. Regardless of addition of metformin, when it is used with insulin lipro, the reduction of HbA1c for dulaglutide 1.5 mg and 0.75 mg were 1.64% and 1.59% respectively. Both dulaglutide doses reduces HbA1c substantially compared to metformin sitagliptin, exenatide twice daily as well as insulin glargine at 26 weeks [38]. In comparison with liraglutide, dulaglutide significantly reduced the HbA1c where the LS mean differences in HbA1c from baseline for dulaglutide was −1.45% and −1.21% for liraglutide in OAM-naïve patients who only carried out the diet therapy [41]. While in patients who were formerly OAM-treated, the differences of LS mean in HbA1c from baseline were −1.35% for dulaglutide and −1.26 for liraglutide. However, both treatment (dulaglutide and liraglutide) has similar percentage of patient achieved HbA1c < 7.0% and <6.5% [41,43]. HbA1c changes from baseline to 52 weeks for dulaglutide 1.5 mg was −1.10%, dulaglutide 0.75 mg was −0.87% and for sitagliptin was 0.39% [46]. Similar trends were observed during the 104th week where the differences from baseline to 104 weeks were −0.99% for dulaglutide 1.5 mg, −0.71% for dulaglutide 0.75 mg and −0.32% for sitagliptin which indicates both dose of dulaglutide were superior than sitagliptin [44]. Significantly greater percentage of participants achieved HbA1c < 7.0% in dulaglutide compared with sitagliptin at both 52 weeks and 104 weeks [44,46].

Dulaglutide reduced fasting serum glucose (FSG) from baseline (at 24 weeks) by −1.70 mmol/L (placebo 0.16 mmol/L), and this reduction was also noted at weeks 12, 24 and 26 [39,43,45]. In addition, baseline seven-point self-monitoring blood glucose (SMBG) profile values were significantly decreased at 24 and 26 weeks compared with placebo [39,43]. In comparison with liraglutide, dulaglutide treatment had given rise to similar least square (LS) mean decreases from baseline in each SMBG value at 26 weeks while significantly reducing values of seven-point SMBG at 52 weeks [41,43]. LS mean change from baseline in FSG for dulaglutide at week 52 was −2.16 mmol/L whereas that of liraglutide was −2.06 mmol/L. At 26 weeks, similar decreases in FSG from baseline in dulaglutide and glargine treatment groups was noted where the LS mean changes were −1.9 mmol/L for dulaglutide and −2.1 mol/L for glargine. The values of SMBG for all time points were significantly reduced in the Dulaglutide group compared with the glargine group except for pre-breakfast [42]. Moreover, in a randomized, phase 3 study, there was a larger reduction in mean FSG from baseline in the two dulaglutide doses (1.5 mg and 0.75 mg) at 52 weeks and 104 weeks compared with sitagliptin [44,46]. LS mean changes from baseline to 104 weeks were −2.0 mmol/L, −1.4 mmol/L and −0.5 mmol/L, respectively.

Dose and background therapy were shown to have effects on the extent of weight changes [38]. The LSM weight change for dulaglutide was −0.91 kg and for placebo was −0.24 kg from baseline to 24 weeks [39]. The difference between groups was not significant. There was a greater weight loss in dulaglutide 1.5 mg and there were similarities in the 0.75 mg versus sitagliptin which were 2.88 ± 0.25, 2.39 ± 0.26 as well as 1.75 ± 0.25 kg, correspondingly [44]. The greatest weight reduction was observed when dulaglutide was added to background metformin where 24–34 patients experienced >5% weight loss compared to dulaglutide monotherapy or other background therapies [38]. The difference in body weight from baseline was not clinically significant in dulaglutide treatment [41,43].

#### 3.2.7. Lixisenatide

A phase 3, randomized controlled trial (GetGoal-F1 study) confirms that both one-step (i.e., 10 μg once daily for 2 weeks followed by 20 μg once daily) and two-step dose-increase regimens (i.e., 10 μg once daily for 1 week, 15 μg once daily for 1 week, followed by the maintenance dose of 20 μg once daily of lixenatide) are comparable in terms of efficacy and tolerability, therefore the simplified one-step dose-increase regimen is recommended for once-daily lixisenatide monotherapy [74]. Furthermore, the marginally lower frequency of AEs such as nausea, vomiting and hypoglycemia and slightly greater in HbA1c reductions with the one-step regimen and this has further promoted the use of one-step regimen. These results are also consistent with a 12-week, phase 3 study of lixisenatide monotherapy in drug-naive T2DM patients (GetGoal-Mono study) [75]. In addition, a one-step regimen is more manageable for patients and physicians, and has similar efficacy and safety profiles as the two-step dose-increase regimen [74,75]. In another 24-week, phase 3 randomized controlled trial, the addition of once-daily lixisenatide in patients who are insufficiently controlled on sulphonylureas, with or without metformin, resulted in significant improvement of blood glucose control, with prominent reduction in PPG levels, without imparting a significant increase in hypoglycemia risk and reduction in body weight as compared with placebo [76]. This is also supported by a pharmacodynamic trial which involves Japanese and Caucasian patients where the Japanese patients were shown to be more responsive to lixisenatide in reducing PPG excursions [77].

In addition to metformin, greater reductions in HbA1C from baseline to week 24 were reported in treatment with the combination of lixisenatide and insulin glargine (LixiLan) compared with insulin glargine alone. The LS mean difference of −0.17 between the mean reduction in glycosylated hemoglobin from baseline for LixiLan and glargine indicating statistical superiority for LixiLan treatment. Besides, the significantly greater reduction of 2-h PPG and 2-h glucose excursion from baseline proved that it also improved PPG control remarkably [78]. Furthermore, a marked decrease in average seven-point glucose profile was shown in patients treated with LixiLan. In short, the composite end points of HbA1C < 7% without weight gain and increase in hypoglycemic risk were met by a greater proportion of patients treated with LixiLan. These findings are also consistent with the previous GetGoal-L and GetGoal Duo-1 trials [78,79,80]. With the approval from FDA in 2016, Soliqua 100/33 is marketed worldwide as a fixed-ratio combination of 100 units long-acting human insulin analog insulin glargine (Lantus) and the 33 mcg GLP-1 RA lixisenatide (Adlyxin). It is indicated for better glycemic control in adults with T2DM insufficiently controlled on basal insulin (less than 60 units daily) or lixisenatide. The clinical rationale for this combination is based on the complementary effects of the individual therapies in which basal insulin improves FPG, while the short-acting lixisenatide decreases PPG [78].

Researchers have shed light on the effect of lixisenatide administration timing on glycemic control based on many clinical trials that administer lixisenatide at pre-breakfast. Studies showed that lixisenatide before main meals has achieved similar reduction in HbA1c compared to lixisenatide before breakfast in patients with insufficient control on metformin. This gives patients the flexibility in dose timing to administer the medicine at their convenience without compromising the efficacy of lixisenatide, thus resulting in better compliance and acceptability [49]. Besides, between the groups, comparable results were obtained regarding weight loss, improvement in average glucose profiles, reduction in FPG and changes in Diabetes Treatment Satisfaction Questionnaire status (DTSQs) score [49]. This is aligned with the GetGoal-M study results of achieving significant improvement in glycemic control after 24-week treatment of lixisenatide added to metformin, regardless of morning or evening meal administration [81]. Comparing the efficacy and safety of lixisenatide with exenatide (a twice-daily short-acting GLP-1RA), GetGoal-X study demonstrated that lixisenatide once-daily is non-inferior to exenatide in controlling HbA1C but extra benefits of achieving greater weight loss and lesser risk of hypoglycemia in patients on metformin but with uncontrolled T2DM.

In comparison with liraglutide, a longer-acting GLP-1 RA, a pharmacodynamics trial showed that lixisenatide 20 μg once-daily provided significantly greater reduction in plasma blood glucose compared to liraglutide [82]. In addition, more lixisenatide-treated patients had reached 2-h PPG levels <140 mg/dL by the more prominent effect of shorter-acting lixisenatide on slowing gastric emptying. Lixisenatide also provided a significant decrease in postprandial insulin, C-peptide and glucagon, and better gastrointestinal tolerability than liraglutide. On the other hand, the longer-acting GLP-1 RA liraglutide provided greater changes in FPG [76,82]. Ultimately, the application of lixisenatide once-daily dosing in clinical settings will be largely dependent on the individual’s preference.

#### 3.2.8. Semaglutide

The efficacy and safety of semaglutide were evaluated by the SUSTAIN clinical trial program [55,83,84]. In a double-blinded, randomized, parallel group and placebo-controlled phase 3a clinical trial programme, T2DM patients who were 18 years or older, and treatment-naive where provided only diet and exercises alone for minimum one month before screening were enrolled [84]. The patients were administered subcutaneous injections of semaglutide at 0.5 mg or 1.9 mg once weekly or volume-matched placebo by using a prefilled dosing pen. Enrolled patients were encouraged to inject semaglutide themselves in the same area of their body at the same day every week for up to 30 weeks [84]. The primary efficacy end-point was observed for 30 weeks based on the changes in mean HbA1c from baseline. The second efficacy end-point was measured as the changes in body weight from baseline for up to 30 weeks [55,83,84]. The mean HbA1c was significantly reduced by 1.45% at week 30 in the 0.5 mg semaglutide treatment group and 1.55% in the 1.0 mg semaglutide treatment group [84]. Changes in body weight were also observed with a significant reduction by 3.73 kg and 4.53 kg in the treatment group of 0.5 mg and 1.0 mg semaglutide, respectively. No significant changes of body weight were reported in placebo group [85,86]. Besides, the placebo group reported that additional antihyperglycemic agents are required to control blood sugar [84]. It is evident that semaglutide reduces the HbA1c significantly and it has a sustained effect compared to the placebo group [83,84].

Among the class of SGLT-2 inhibitors, dapagliflozin was found to be the most efficacious antidiabetic agent to improve glycemic control with a reduction of 1.1% of HbA1c at a given dose of 10 mg. As for GLP-1 agonists, semaglutide seems to be the most efficacious antidiabetic agent compared to other drugs in the same class, with a reduction of HbA1c by 1.6% at a given dose of 1.0 mg. In the class of DPP-4 inhibitor, alogliptin was found to reduce HbA1c by 0.5 ± 0.7%. In the overall comparison, semaglutide under the class of GLP-1 agonists was identified to be the most efficacious antidiabetic agent with the highest reduction of HbA1c when compared to the other classes of medication.

### 3.3. Safety

#### 3.3.1. Canagliflozin

Canagliflozin was well tolerated by patients as shown in many studies, without serious AEs [12,13,14,15,16,56,58,60,61,63,64,65,85]. The majority of the AEs reported were of mild to moderate severity. There was no incidence of death reported during the studies [64]. The most common AEs reported for canagliflozin are irritation over ECG electrode application site and skin-related AEs such as erythema, lichenification, pruritus, psoriasis, and skin irritation [56]. Vaginal candidiasis was also reported as one of the AEs that occurred during the study, which is consistent with the finding that SGLT-2 inhibitors led to an increase in the occurrence of genital mycotic infections [13,14,15,16,56,57,58,59,60,63]. However, factors such as prior history of genital mycotic infection or being uncircumcised were considered for the increased occurrence of genital mycotic infections [58]. Patients treated with canagliflozin were also associated with higher incidence of osmotic diuresis and polyuria [12,13,15,16,57,58,60,61,62,63]. The incidence of hypoglycemia was slightly higher in patients on canagliflozin and the events occurred mostly in the morning [12,13,14,15,56,57,60]. Hence, caution should be given when the patient is receiving a combination of canagliflozin and insulin in the morning, but the hypoglycemic events could be prevented by adjusting the dose of insulin [12]. Canagliflozin should be given to patients older than 75 years old with caution as the incidence of AEs were higher in this population [59].

#### 3.3.2. Alogliptin

There were 36 treatment-emergent adverse events (TEAEs) noted in a study of 20 subjects of a group of children, teenagers, and adults with T2DM. Most of the TEAEs were mild with Alogliptin and the most commonly reported AEs were headache, abdominal pain, fatigue and nausea. However, some changes in blood biochemistry were noted such as increased blood creatine kinase, decreased neutrophil count, decreased hematocrit and haemoglobin. There were no AEs related to drug discontinuation or deaths among the subjects [18]. In another study, all treatment groups reported similar overall frequencies of AEs where alogliptin alone showed incidences of 57.7% (41/71), alogliptin/metformin once daily with 50.7% (77/152) of incidences, and 52.3% (79/151) with alogliptin/metformin twice daily doses [17]. Nasopharyngitis was the most common AE across all treatment groups, however, the incidence of serious hypoglycemia or acute pancreatitis were absent in the combination therapy of metformin and alogliptin [17].

#### 3.3.3. Dapagliflozin

Several studies showed that dapagliflozin is generally well-tolerated. A slightly higher overall AE was observed with dapagliflozin treatment groups. The most common AEs in patients treated with dapagliflozin were nasopharyngitis [23], genital infection or UTI [25,27]. The infection is potentially due to changes in immune function and presence of glycosuria. A long-term study showed that the group treated with dapagliflozin experienced significant volume depletion [70]; this may be possibly due to the diuretic properties of dapagliflozin [23,70]. Besides, a combination therapy study done by Eiichi Araki, et al. showed that dapagliflozin may increase hematocrit and hemoglobin levels due to decreased plasma volume [25]. Episodes of hypoglycaemia and renal impairment are uncommon in patients treated with dapagliflozin with no incidences of severe hypoglycemia and renal failure, and no events lead to discontinuation [23,25,27]. In some cases, elevations of total bilirubin and liver enzymes (aspartate aminotransferase and alanine aminotransferase) were also reported [69]. Although it is rare, a study by Lambers Heerspink H. J., et al. reported dizziness and syncope related to dapagliflozin treatment [70]. 

#### 3.3.4. Empagliflozin

A long-term treatment of T2DM with empagliflozin showed that there was a small increment in hematocrit and β-hydroxybutyrate levels and also small reduction in uric acid and Glomerular Filtration Rate (GFR) [31,86]. It is likely to resulted from the hemodynamic changes attributed to the effects on tubular feedback mechanisms [28,30]. Treatment with empagliflozin does not promote glomerular or tubular damage as the concentration of markers such as urinary albumin and α-1 microglobulin were not elevated [86]. Furthermore, treatment with empagliflozin was also accompanied with small changes in patients’ lipid profiles [86]. Small increases in low density lipoprotein (LDL) cholesterol levels were observed in the dose of 10 mg and 25 mg of empagliflozin where small increment of total cholesterol was noted in the 10 mg of empagliflozin [31]. In addition, high density lipoprotein (HDL) cholesterol level was significantly elevated and triglycerides (TG) were significantly reduced in both groups. There was also presence of significant changes in baseline free fatty acid levels with the 25-mg of empagliflozin group compared with placebo [32]. This showed that empagliflozin could lead to reduced body weight that may be due to the empagliflozin-induced urinary glucose excretion. It results in decreasing glucose levels in plasma with an increased glucagon-to-insulin ratio by causing lipolysis, increased free fatty acid levels and leads to ketogenesis [30,32]. There were no significant changes in electrolyte levels such as potassium, phosphate, calcium, sodium and magnesium level in any treatment group after 78-week of extension trial [32,86]. No severe AEs were reported that may lead to empagliflozin withdrawal from treatment. Genital infection-bartholinitis was reported in patients with empagliflozin [32] and this condition is usually more common in female than male patients [28,31].

#### 3.3.5. Albiglutide

The most common AEs reported with albiglutide were injection-site reactions (ISRs), gastrointestinal reactions at a dose of 30 mg and 50 mg compared to placebo [72,73]. The other common AEs reported frequently in albiglutide treatment group were symptomatic hypoglycemia, nausea, vomiting and diarrhea [73]. Statistics shown nausea with or without vomiting is the most common reported event with 29% of subjects in albiglutide 30 mg and 54.3% in albiglutide 50 mg, respectively. As for GI-related events, hypoglycemia was not increased with albiglutide to placebo and exenatide as shown in the study [72]. However, most of the events were mild and no withdrawal from the study was required [72,73].

#### 3.3.6. Dulaglutide

Similar to other GLP-1 receptor agonists, dulaglutide therapy may lead to gastrointestinal AEs, particularly transient and mild to moderate intensity of nausea, vomiting, diarrhea and eructation [39]. The three phase 3 studies revealed that 0.75 mg of dulaglutide once weekly through week 26 produced nausea in an overall 7.1% of total 855 patients. The incidence of nausea was significantly different between the subgroup for their gender, body weight, duration of diabetes and concomitant use of sulfonyl ureas (SU). Nausea and vomiting were more common with dulaglutide than sitagliptin and placebo [87]. Patient taking dulaglutide exhibited risk of hypoglycemia. The incidence of total and nocturnal hypoglycemia was higher (22.1%) in elderly patients with low body mass index (>65 years, <25 kg/m^2^) among four subgroups combining low and high age and body mass groups through 26 weeks [37,40]. In another study, the incidence of total hypoglycemia for dulaglutide 1.5 mg, dulaglutide 0.75 mg and sitagliptin at 52 weeks were 10.2%, 5.3% and 4.8%, respectively, and 12.8%, 8.6% and 8.6%, respectively, at 104 weeks [44,46].

Increases in serum lipase and amylase were also common problems in dulaglutide-treated patients [39]. However, compared to the dulaglutide treatment group, liraglutide-treated patients have significantly increased postbaseline lipase level above the upper limit of normal (ULN) with median increases of 9.0 U/L and 6.0 U/L, respectively. While in terms of postbaseline amylase, no significant different was observed between liraglutide and dulaglutide [41,43]. Moreover, both dulaglutide doses of 0.75 mg and 1.5 mg also gave a greater elevation of serum lipase, and total amylase and pancreatic amylase levels were within the normal range when compared with sitagliptin [44,46]. The safety of dulaglutide was assessed in a phase III, randomized, double-blind, placebo-controlled study for the comparison of 24-week once-weekly dulaglutide 1.5 mg with placebo [39]. The study revealed that 3.8% of the dulaglutide treatment group experienced serious AEs including anaplastic astrocytoma, angina pectoris, cerebrovascular accident, hypoglycemia, osteomyelitis, otitis media, pulmonary tuberculosis, rheumatoid arthritis and ulna fracture. Fatal reaction was reported with one death of the participant randomized to the dulaglutide treatment group who discontinued the study due to enterocolitis [39]. In another study, a participant receiving dulaglutide 1.5 mg experienced stroke and died [44].

#### 3.3.7. Lixisenatide

Consistent with other GLP-1 receptor agonists, the most commonly reported AEs associated with lixisenatide are gastrointestinal in nature, predominantly nausea, followed by vomiting and diarrhea [88]. The incidence of AEs was usually observed in the early phase of treatment and decreased over the span of treatment, as reported in GetGoal-Mono, GetGoal-Mono-Japan and the GetGoal-L-Asia studies [88]. The incidence of nausea in lixisenatide monotherapy was approximately 23% and placebo 4.1% while incidence of symptomatic hypoglycemia was comparable in both lixisentide and placebo patients, with no severe episodes [75]. The GetGoal-Mono-Japan study found that once-daily lixisenatide monotherapy was well-tolerated in Japanese patients with T2DM for long-term treatment. Throughout the entire treatment period of 76 weeks, this intervention was shown to have consistent efficacy, safety and tolerability in addition to low frequency of serious treatment-emergent AEs with an incidence frequency of 4.3% [88].

#### 3.3.8. Semaglutide

The AEs reported in the use of semaglutide were mostly gastrointestinal related such as nausea and diarrhea. Nausea was reported as 20% and 24% in 0.5 mg and 1.0 mg semaglutide treatment groups, respectively, while diarrhea was reported as 13% and 11% in 0.5 mg and 1.0 mg semaglutide treatment group [84]. However, the severity of most of the AEs reported were mild to moderate and the frequency of the serious AEs were less in the semaglutide group [55,83,84]. Semaglutide also had beneficial effects on cardiovascular outcomes which decrease the risk of non-fatal stroke and myocardial infarction [54]. As for the microvascular complication, these studies show that semaglutide had similar safety profiles to other GLP-1 receptor agonists [54,55,83,84]. The detailed comparison of the antidiabetic drug efficacy and safety are presented in Table 1.

Canagliflozin, dapagliflozin and empagliflozin are SGLT-2 inhibitors that have been commonly associated with an upsurge incidence of genital mycotic infections. Among all three SGLT-2 inhibitors, canagliflozin was found to have the lowest incidence (39.8% at week 26) of AEs. GLP-1 agonists, albiglutide, semaglutide, lixisenatide and dulaglutide are well tolerated by patients, and most of the AEs of this group of drugs were gastrointestinal related, with more cases of injection site reactions reported. Among the GLP-1 agonists, semaglutide was the safest agent with the lowest incidence (56% at week 30) of AEs. However, when comparing all the eight agents, alogliptin, under the class of DPP-4 inhibitor, has the lowest incidence rate of AEs (4% at week 24), making it the safest agent among the eight agents approved in the past five years.

This systematic review found that the primary efficacy endpoint for most randomized controlled trials of antidiabetic drugs was the reduction of HbA1c levels from baseline. Among the eight novel medications marketed from 2013 to 2017, once-weekly semaglutide has demonstrated the highest efficacy. As shown in the 56-week, SUSTAIN 2 trials, baseline HbA1c was significantly reduced by 1.3% in the semaglutide 0.5-mg treatment arm and 1.6% HbA1c level from baseline at the dose of 1.0 mg. This result is consistent with other findings, namely, SUSTAIN 1 and SUSTAIN 3. In addition, dulaglutide which is also categorized as the GLP-1 receptor agonists has although lower but remarkable efficacy at endpoint as semaglutide. A 52-week Award-5 trial showed that semaglutide 0.75 mg reduces baseline HbA1c by 0.87% while 1.1% baseline HbA1c reduction is achieved by dulaglutide.

In terms of safety, alogliptin is known to be relatively safe as it has similar and less overall frequency of AEs compared to other treatment groups. There are several drugs that also have similar safety profiles compared to placebo or other treatment groups, but have AEs reported in the treated group. Empagliflozin has reports of urinary tract infections whereas canagliflozin has an increased incidence of genital mycotic infections. Albiglutide and semaglutide are more commonly reported with gastrointestinal AEs such as nausea and diarrhea. Lixisenatide and Dulaglutide have slightly higher percentages of symptomatic events, but severe events are rare.

## 4. Conclusions

In summary, this review provides a comprehensive introduction about the efficacy and safety of the eight antidiabetic agents that were approved and introduced in to the market from 2013 to 2017. Based on the outcome assessment comparison, while all the agents demonstrated favorable efficacies in lowering HbA1c, semaglutide was identified to be the most efficacious agent among the eight agents approved in the recent five years. In the studies, all the agents were tolerated well with similar safety profiles. However, further and larger trials are warranted to establish the long-term safety of these agents.

## Figures and Tables

**Table 1:** Summary of study characteristics and results in HbA1c reduction and safety corresponding to eight FDA-approved antidiabetic drugs from 2013 to 2017.

Drug Name	Author, Year, [Reference Number]	Study Design	Population Characteristic	Interventions	Primary End Points	Results	
						Results in HbA1c Reduction	Safety
Canagliflozin	Inagaki, N., et al., 2016. [12]	Randomized, parallel-group, double-blind study.	Patients who had inadequate glycemic control despite insulin, diet and exercise therapies	Canagliflozin 100 mg, placebo.	The change in glycated hemoglobin (HbA1c) levels from the baseline to week 16.	Week 16: −0.97% (100 mg)	**AEs**: 64.8% **Major AEs**: Decreased blood glucose, hypoglycemia, pollakiuria, and polyuria **Hypoglycemia**: 40%
	Rodbard, H. W., et al., 2016. [14]	Randomized, double-blind, parallel-group, multicenter study was conducted at 47 study centres in five countries.	Patients with T2DM on metformin ≥1500 mg/day and sitagliptin 100 mg	Canagliflozin 100 mg (increased to 300 mg after 6 weeks), placebo	Change from baseline in HbA1c at week 26.	Week 26: −0.91% (pooled 100 mg and 300 mg)	**AEs**: 39.8% **Major AEs**: Genital mycotic infections. **Hypoglycemia**: 3.7%
	Forst, T., et al., 2014. [63]	This randomized, double-blind, placebo- and active-controlled, phase 3 study was conducted at 74 centres in 11 countries.	Patients with type 2 diabetes mellitus (T2DM) inadequately controlled with metformin and pioglitazone.	Canagliflozin 100 mg, Canagliflozin 300 mg, placebo.	Hemoglobin A1c (HbA1c), body weight fasting plasma glucose and systolic blood pressure.	Week 26: −0.89% (100 mg); −1.03% (300 mg)	**AEs**: 69.9% (100 mg); 76.5% (300 mg) **Major AEs**: Genital mycotic infections, AEs related to osmotic diuresis and volume depletion. **Hypoglycemia**: 4.4% (100 mg); 6.1% (30.0 mg)
	Wilding, J. P. H., et al., 2013. [56]	Randomized, double-blind, placebo-controlled, Phase 3 study conducted at 85 study centres in 11 countries.	Eligible patients were men and women aged 18–80 years with T2DM who had inadequate glycemic control (HbA1c ≥ 7.0% to ≤10.5%) on metformin plus sulphonylurea, with both agents at maximally or near-maximally effective doses.	Canagliflozin 100 mg, Canagliflozin 300 mg, placebo.	Change in HbA1c at 26 weeks.	Week 26: −0.85% (100 mg); −1.06% (300 mg) Week 52: −0.74% (100 mg); −0.96% (300 mg)	**AEs**: <4% **Major AEs**: Genital mycotic infections, osmotic diuresis-related AEs. **Hypoglycemia**: 33.8% (100 mg); 36.5% (300 mg)
	Lavalle-González, F. J., et al., 2013. [57]	Randomized, double-blind, placebo- and active-controlled, Phase 3 study conducted at 169 centres in 22 countries.	Men and women with type 2 diabetes, aged ≥18 and ≤80 years, who had inadequate glycemic control and who were on stable metformin therapy	Canagliflozin 100 mg, Canagliflozin 300 mg, Sitagliptin 100 mg, placebo.	Change from baseline in HbA1c at week 26;	Week 26: −0.79% (100 mg); −0.94% (300 mg) Week 52: −0.73% (100 mg); −0.88% (300 mg)	**AEs**: 72.3% (100 mg); 62.7% (300 mg) **Major AEs**: Genital mycotic infection, osmotic diuresis-related AEs. **Hypoglycemia**: 6.8%
Alogliptin	Kohei Kaku, et al., 2017. [17]	Phase III, randomized, double-blind, parallel-group, multicenter study	Mean age 57.2, mean diabetes duration 7.16 years, mean HbA1c 7.84%	Alogliptin (25 mg once daily), alone or with metformin hydrochloride (500 mg once daily or 250 mg twice daily	Change in glycated hemoglobin (HbA1c) from baseline to the end of treatment (week 24)	Week 24: Alogliptin alone: 0.16 (0.072) % Alogliptin/metformin once daily: −0.49 (0.049)% Alogliptin/metformin twice daily: −0.60 (0.049)%	**AEs**: 57.7% (Alogliptin alone); 50.7% (Alogliptin/metformin once daily); 52.3% (Alogliptin/metformin twice daily) **Major AEs**: Nasopharyngitis, pharyngitis, hypoesthesia **Hypoglycemia**: No patients developed serious hypoglycemia
	Tanaka, K., et al., 2017. [66]	Prospective randomized open-label study	**Study 1**: DPP-4 inhibitor-naive Alogliptin: mean age 63.6 years, mean duration of diabetes 9.8 years, mean HbA1c 7.2% Vildagliptin: Mean age 65.8 years, mean duration of diabetes 8.0 years, mean HbA1c 7.5%	**Study 1**: Alogliptin 25 mg once daily, Vildagliptin 50 mg twice daily	The change in HbA1c levels at 24 weeks.	Week 24: **In Study 1**, Alogliptin group: 0.5 ± 0.7% (*p* = 0.002) Vildagliptin group: -0.7 ± 0.9% (*p* = 0.001)	**AEs**: **In Study 1**: 4.0% (Alogliptin): 0% (Vildagliptin)
			**Study 2**: T2DM on treatment with 50 mg/day sitagliptin Alogliptin: Mean age 66.7 years, mean duration of diabetes 10.8 years, mean HbA1c 6.8%Vildagliptin: Mean age 66.2 years, mean duration of diabetes 11.7 years, mean HbA1c 7.0%	**Study 2**: Sitagliptin 50 mg once daily switch to alogliptin 25 mg once daily, Sitagliptin 50 mg once daily switch to vildagliptin 50 mg twice daily		**In Study 2**, switch-to-alogliptin group: 0.2 ± 0.7% (*p* = 0.007), switch-to-vildagliptin group: 0.0 ± 0.6 (*p* = 0.188),	**In Study 2**: Switch-to-alogliptin group: 1.6%Switch-to-vildagliptin group: 2.9% **Major AEs**: - **Hypoglycemia**: All patients developed hypoglycemia
Dapagliflozin	Eiichi Araki, et al., 2016. [25]	Multicenter, randomized, double-blind, parallel-group, placebo-controlled study	Mean age: 58.0, mean duration of diabetes: 14.97 years, mean HbA1c 8.34%	Dapagliflozin 5 mg plus metformin therapy, placebo plus metformin therapy	The primary efficacy end-point was the change in hemoglobin A1c (HbA1c) from baseline at week 16	Week 16: −0.55%	**AEs**: 48.8% **Major AEs**: Nasopharyngitis, pollakiuria, thirst **Hypoglycemia**: 19.5%
	William, T. Cefalu, et al., 2015. [24]	Multicenter, randomized, double-blind, placebo-controlled, international, phase 3 study	Dapagliflozin group: mean age 62.8 years, mean duration of diabetes 12.6 years, mean HbA1c 8.18%. Placebo group: Mean age 63.0, mean duration of diabetes 12.3 years, mean HbA1c 8.08%	Dapagliflozin 10 mg, placebo	Co-primary end points were a change from baseline in hemoglobin A_1c_ (HbA1c) and the proportion of patients achieving a combined reduction in HbA1c of ≥0.5% (5.5 mmol/mole), body weight (BW) of ≥3%, and systolic blood pressure (SBP) of ≥3 mmHg.	Week 24: −0.38%	**AEs**: 73.9% **Major AEs**: Cardiac disorder, dizziness, nasopharyngitis **Hypoglycemia**: 25.2%
	Linong, Ji, et al., 2014. [27]	Phase III, multicenter, parallel-group, double-blind study	Dapagliflozin 5 mg group: Mean age (years) 53, mean duration of diabetes (years) 1.15, mean HbA1c (%) 8.14. Dapagliflozin 10 mg group: Mean age (years) 51.2, mean duration of diabetes (year) 1.67, mean HbA1c (%) 8.28. Placebo group: mean age (years) 49.9, mean duration of diabetes (years) 1.30, mean HbA1c (%) 8.35.	Placebo, dapagliflozin 5 mg, dapagliflozin 10 mg	Change in glycosylated hemoglobin (HbA1c) at week 24	Week 24: −1.04% (5 mg); −1.11% (10 mg)	**AEs**: 61.7% (5 mg); 60.9% (10 mg) **Major AEs**: Nasopharyngitis, urinary tract infection **Hypoglycemia**: 0.8% (5 mg); 0.8% (10 mg)
Empagliflozin	J. Rosenstock, et al., 2015. [28]	Randomized, placebo-controlled, double-blind phase IIb study	HbA1c > 7 to ≤10% (>53 to ≤86 mmol/mole)] on basal insulin (glargine, detemir, NPH)	Empagliflozin 10 mg, empagliflozin 25 mg, placebo	Change from baseline in HbA1c at week 18.	Week 18: −0.6 % (10 mg); −0.7 % (25 mg) Week 78: −0.5% (10 mg); −0.6% (25 mg)	**AEs** (at week 78): 85% (10 mg); 87% (25 mg)Major AEs: Hypoglycemia, nasopharyngitis, urinary tract infection **Hypoglycemia**: Week 18: 20% (10 mg); 28% (25 mg)Week 78: 36% (10 mg); 36% (25 mg)
	Michael Roden, et al., 2015. [31]	Phase III, parallel-group, randomized, double-blind trial	Mean age 55 years, mean HbA1c: 7.88%	Empagliflozin 10 mg, Empagliflozin 25 mg, placebo, sitagliptin 100 mg	Exploratory endpoints included changes from baseline in HbA1c, weight and blood pressure at week 76	Week 76: −0.65% (10 mg); −0.76% (25 mg)	**AEs**: 76.8% (10 mg); 78% (25 mg) **Major AEs**: Hyperglycemia, nasopharyngitis, urinary tract infection **Hypoglycemia**: 0.9% (10 mg); 0.9% (25 mg)
Albiglutide	Rosenstock, J., et al., 2009. [35]	Phase II trial, randomized double-blind placebo-controlled parallel-group study conducted at 118 sites in the U.S.	Mean age 54 years, diabetes duration 4.9 years, HbA1c 8.0%	Subcutaneous placebo or albiglutide (weekly [4, 15, or 30 mg], biweekly [15, 30, or 50 mg], or monthly [50 or 100 mg]) or exenatide twice daily.	Change from baseline HbA1c of albiglutide groups versus placebo at week 16.	Week 16: −0.87% (30 mg weekly); −0.79% (50 mg biweekly); −0.87% (100 mg monthly)	**AEs**: 67–85% **Major AEs**: Nausea, vomiting, headache **Hypoglycemia**: 0–3.1%
	Michael, A. Nauck, et al., 2016. [73]	3-year study with four study periods: screening (2 weeks); run-in (4 weeks); treatment (156 weeks, comprising 52 weeks for primary endpoint) and post-treatment follow-up (8 weeks)., randomized, placebo-controlled study.	Age ≥ 18 years, with type 2 diabetes uncontrolled by diet and exercise, not using a glucose-lowering agent, HbA1c 7.0–10.0%.	Albiglutide 30 mg, Albiglutide 50 mg, placebo.	Change in HbA1c from baseline to week 52.	Week 52: −0.84% (30 mg); −1.04% (50 mg)	**AEs**: 78.2% (30 mg); 81.8% (50 mg) **Major AEs**: nausea, diarrhea, vomiting, injection-site reaction **Hypoglycemia**: 5.9% (30 mg); 6.1% (50 mg)
Dulaglutide	Dungan, K. M. et al., 2016. [39]	24-week, multicenter, randomized, double-blind, placebo-controlled	T2DM patients inadequately controlled on sulphonylurea. Mean age 58; mean diabetes duration 7.6 years; mean HbA1c 8.4%.	Dulaglutide 1.5 mg, placebo	HbA1c change from baseline at 24 weeks	Week 24: −1.4%	**AEs**: 46.4% **Major AEs**: Nausea, diarrhea, eructation **Hypoglycemia**: 20.9%
	Nauck, M. et al., 2014. [46]	52-week, randomized, multicenter, double-blind trial	T2DM patients inadequately controlled on metformin. Mean age 54; mean diabetes duration 7 years; mean HbA1c 8.1%	Dulaglutide 1.5 mg, dulaglutide 0.75 mg, sitagliptin 100 mg	Change in HbA1c concentration ay 52 weeks from baseline.	Week 52: −1.10 ± 0.06% (1.5 mg); −0.87 ± 0.06% (0.75 mg)	**AEs**: 77% (1.5 mg); 77% (0.75 mg) **Major AEs**: Gastrointestinal disorders, decreased appetite **Hypoglycemia**: 10.2% (1.5 mg); 5.3% (0.75 mg)
	Miyagawa, J. et al., 2015. [43]	Phase III, 52-week (26-week primary endpoint), randomized, double-blind, placebo-controlled, open-label comparator (liraglutide) trial	T2DM Japanese patients. Mean age 57.4; mean diabetes duration 6.6 years; mean HbA1c 8.14%	Dulaglutide 0.75 mg, liraglutide 0.9 mg, placebo	Superiority of dulaglutide versus placebo on change from baseline in HbA1c at 26 weeks	Week 26: −1.43%	**AEs**: 56.1% **Major AEs**: Nasopharyngitis, decreased appetite, gastrointestinal disorders **Hypoglycemia**: 2.1%
Lixisenatide	Rosenstock et al., 2014. [76]	24-week, randomized, double-blind, placebo-controlled, parallel-group, multicenter	T2DM treated with sulphonylurea ± metformin. Mean age 57; mean diabetes duration 9.4 years; mean HbA1c 8.25%	Lixisenatide 20 μg once-daily in a stepwise dose increase, placebo	Change in HbA1c from baseline to week 24	Week 26: −0.85%	**AEs**: 68.3% **Major AEs**: Gastrointestinal disorders, mostly nausea **Hypoglycemia**: 15.3%
	Bolli, G. B. et al., 2014. [74]	24-week and ≥52-week variable extension period, randomized, double-blind, placebo-controlled, parallel-group, multi-center	T2DM treated with metformin. Mean age 56; mean diabetes duration 6 years; mean HbA1c 8%	Lixisenatide one-step dose increase, lixisenatide two-step dose increase, placebo	HbA1c reduction at week 24	Week 24: −0.90 ± 0.1% (one-step); −0.80 ± 0.10% (two-step)	**AEs**: 67.7% (one-step); 70.8% (two-step) **Major AEs**: Gastrointestinal disorders, mostly nausea, vomiting **Hypoglycemia**: 1.9% (one-step); 2.5% (two-step)
	Riddle, M. C. et al., 2013. [79]	24-week, randomized, double-blind, placebo-controlled	T2DM with established basal insulin therapy but inadequate glycemic control. Mean age 57; mean diabetes duration 12.5 years; mean HbA1c 8.4%; mean duration of basal insulin uses 3.1 years	Lixisenatide 20 μg once-daily, placebo	HbA1c reduction from baseline	Week 24: −0.7± 0.1%	**AEs**: 68.3% **Major AEs**: Gastrointestinal disorders, mostly nausea, vomiting **Hypoglycemia**: 26.5%
Semaglutide	Ahmann, A. J., et al. 2018. [83]	Phase 3a, open-label, parallel-group, randomized controlled trial.	Subjects with type 2 diabetes taking oral antidiabetic drugs.	Semaglutide 1.0 mg, Exenatide ER 2.0 mg.	Change from baseline in HbA1c at week 56.	Week 56: −1.5%	**AEs**: 41.8% **Major AEs**: Gastrointestinal AEs, injection site reactions. **Hypoglycemia**: Nil
	Sorli C, Harashima S-I, et al., 2017. [55]	Double-blind, randomized, parallel-group, international, placebo-controlled phase 3a trial (SUSTAIN 1) at 72 sites in Canada, Italy, Japan, Mexico, Russia, South Africa, UK, and USA.	Eligible participants were treatment-naive individuals aged 18 years or older with type 2 diabetes treated with only diet and exercise alone for at least 30 days before screening, with a baseline HbA1c of 7.0–10.0% (53–86 mmol/mole).	Once-weekly subcutaneously injected semaglutide (0.5 mg or 1.0 mg), or volume-matched placebo (0.5 mg or 1.0 mg)	The change in mean HbA1c from baseline to week 30	Week 30: −1.45% (0.5 mg); −1.55% (1.0 mg)	**AEs**: 20% (0.5 mg); 24% (1.0 mg) **Major AEs**: Gastrointestinal AEs-nausea, diarrhea. **Hypoglycemia**: Nil
	Ahren B, et al., 2017. [84]	56-week, phase 3a, randomized, double-blind, double-dummy, active-controlled, parallel-group, multinational, multicenter trial (SUSTAIN 2) at 128 sites in 18 countries.	Eligible patients were aged at least 18 years (or at least 20 years in Japan) and diagnosed with type 2 diabetes, with insufficient glycemic control (HbA1c 7.0–10.5% [53.0–91.0 mmol/mole]) despite stable treatment with metformin, thiazolidinediones, or both.	Change in HbA1c from baseline to week 56, assessed in the modified intention-to-treat population (all randomly assigned participants who received at least one dose of study drug);	SC semaglutide 0.5 mg once weekly plus PO sitagliptin placebo OD; SC semaglutide 1.0 mg once weekly plus PO sitagliptin placebo OD, PO sitagliptin 100 mg OD plus SC semaglutide placebo 0.5 mg once weekly, or PO sitagliptin 100 mg OD plus SC semaglutide placebo 1.0 mg once weekly.	**Week 56**: −1.3% (0.5 mg); −1.6% (1.0 mg)	**AEs**: 18% (0.5 mg); 18% (1.0 mg) **Major AEs**: Gastrointestinal AEs-nausea, diarrhea. **Hypoglycemia**: 2% (0.5 mg); <1% (1.0 mg)
